# The Microfluidic Environment Reveals a Hidden Role of Self-Organizing Extracellular Matrix in Hepatic Commitment and Organoid Formation of hiPSCs

**DOI:** 10.1016/j.celrep.2020.108453

**Published:** 2020-12-01

**Authors:** Federica Michielin, Giovanni G. Giobbe, Camilla Luni, Qianjiang Hu, Ida Maroni, Michael R. Orford, Anna Manfredi, Lucio Di Filippo, Anna L. David, Davide Cacchiarelli, Paolo De Coppi, Simon Eaton, Nicola Elvassore

**Affiliations:** 1 Great Ormond Street Institute of Child Health, University College London, WC1N1EH London, UK; 2 Shanghai Institute for Advanced Immunochemical Studies (SIAIS), ShanghaiTech University, 201210 Shanghai, China; 3 Department of Industrial Engineering, University of Padova, 35131 Padova, Italy; 4 Venetian Institute of Molecular Medicine (VIMM), 35129 Padova, Italy; 5 Telethon Institute of Genetics and Medicine (TIGEM), Armenise/Harvard Laboratory of Integrative Genomics, 80078 Pozzuoli, Italy; 6 Next Generation Diagnostic Srl, 80078 Pozzuoli, Italy; 7 Elizabeth Garrett Anderson Institute for Women’s Health, University College London, WC1E 6AU London, UK; 8 Department of Translational Medicine, University of Naples “Federico II,” 80131 Naples, Italy; 9 Specialist Neonatal and Paediatric Surgery, Great Ormond Street Hospital, WC1N 3JH London, UK

**Keywords:** microfluidics, SILAC-MS, proteome analysis, ECM remodeling

## Abstract

The specification of the hepatic identity during human liver development is strictly controlled by extrinsic signals, yet it is still not clear how cells respond to these exogenous signals by activating secretory cascades, which are extremely relevant, especially in 3D self-organizing systems. Here, we investigate how the proteins secreted by human pluripotent stem cells (hPSCs) in response to developmental exogenous signals affect the progression from endoderm to the hepatic lineage, including their competence to generate nascent hepatic organoids. By using microfluidic confined environment and stable isotope labeling with amino acids in cell culture-coupled mass spectrometry (SILAC-MS) quantitative proteomic analysis, we find high abundancy of extracellular matrix (ECM)-associated proteins. Hepatic progenitor cells either derived in microfluidics or exposed to exogenous ECM stimuli show a significantly higher potential of forming hepatic organoids that can be rapidly expanded for several passages and further differentiated into functional hepatocytes. These results prove an additional control over the efficiency of hepatic organoid formation and differentiation for downstream applications.

## Highlights

• Microfluidic confined environment enhances hepatic differentiation of hPSCs
• SILAC-based proteomic analysis reveals high abundance of secreted ECM proteins
• ECM deposition and remodeling correlate with cell-ECM receptor overexpression
• Either endogenous or exogenous ECM enhances organoid formation and differentiation

## Graphical Abstract

**Figure:**
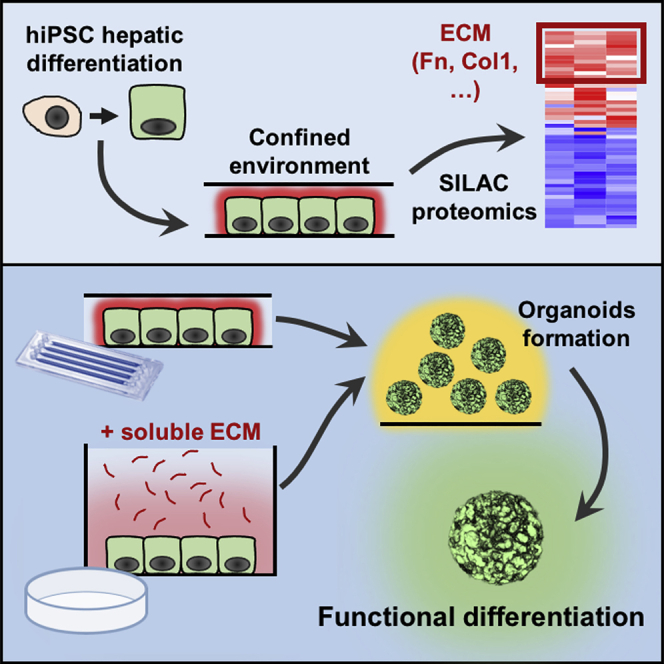

## Teaser

Michielin et al. investigate the secretome of human pluripotent stem cells undergoing hepatic differentiation by coupling microfluidics with SILAC proteomic analysis. They reveal a role of soluble ECM protein accumulation and deposition and leverage these insights to efficiently and robustly derive hepatic organoids from hiPSCs.

## Introduction

The specification of cell identity during mammalian liver development relies on the activity of transcriptional networks. These networks are controlled by extrinsic signals that restrict and define distinct cell fates (Mamidi et al., 2018). Moreover, tightly regulated cellular self-organization programs are mediated by mutual interactions between cells and their extracellular environment, ensuring the robustness of tissue and organ development (Bonnans et al., 2014).

The study of *in vivo* human liver development is restricted by the availability of human liver samples during the initial 6 weeks of gestation. Alternatively, key stages of human liver organogenesis can be recapitulated *in vitro* through the differentiation of human pluripotent stem cells (hPSCs) (Wandzioch and Zaret, 2009).

From mouse *in vivo* studies, it is known that following the formation of the foregut endoderm, fibroblast growth factor (FGF), and bone morphogenetic protein (BMP) signaling from the surrounding mesoderm induce hepatic fate. Shortly after hepatic specification, the epithelium begins to express liver genes (Albumin, Afp, and Hnf4α) and thickens while cells undergo morphological changes giving rise to the pseudostratified liver diverticulum, where hepatoblasts delaminate and migrate into the surrounding mesenchyme to form the nascent 3D-structured liver bud. Concomitantly, a profound remodeling of the extracellular matrix (ECM) involving metalloproteinases Mmp14 and Mmp2 occurs (Si-Tayeb et al., 2010a). In addition to FGF and BMP, hepatocyte growth factor (HGF) signaling from the septum transversum mesenchyme is required at this stage for hepatoblast proliferation and liver bud growth (Zorn, 2008; Si-Tayeb et al., 2010a), whereas the 3D assembly allows for the formation of proper polarity during maturation.

Based on the knowledge of mouse embryo development, differentiation protocols of hPSCs have been optimized. Initially, 2D culture systems have been developed based on the supply of exogenous extrinsic signals, including growth factors and cytokines, mimicking the biochemical signals that activate these specific signaling pathways in a precise dose and temporal scale (Hay et al., 2008; Si-Tayeb et al., 2010b). More recently, the 3D organoid technology has been developed to recapitulate *in vitro* stages of human liver organogenesis from human induced pluripotent stem cells (hiPSCs), particularly related to the early gestational weeks. For instance, ECM gel (Matrigel) has been used to generate hepatic organoids by harnessing the self-organization potential of endodermal cells to form hepatobiliary organoids with enhanced functional features and potency to generate multiple lineages (Guan et al., 2017; Akbari et al., 2019; Ouchi et al., 2019). 3D Matrigel embedding also enhanced hepatic maturation of organoids generated from hepatocyte-like cells derived in 2D (Ng et al., 2018; Mun et al., 2019). Interestingly, decellularized liver scaffolds have been also shown to increase hepatic differentiation by providing 3D structure and cell contact with liver-specific ECM proteins (Wang et al., 2016a). Overall, these studies show that the 3D self-organization is a powerful approach to both recapitulate organogenesis and to derive more functional cells.

Despite these achievements, it is still not clear how hiPSCs, when exposed to the sequential supplementation of exogenous FGF, BMP, and HGF, which mimic key developmental stages both in 2D or 3D approaches, activate a secretory activity involving morphogens, growth factors and cytokines, ECM deposition, and remodeling, all of which can influence and dictate cellular behavior despite the cells' genetic program. This interplay between individual cells (or subsets of cells) and their environment is a continual process with no defined endpoint. In particular, it is still unknown how the secretome contributes to the acquisition of the differentiated phenotype (Wolling et al., 2018; Farina et al., 2011). We hypothesize that the contribution of the hiPSC secretome, as a consequence of the exogenous signals, is key to induce proper hepatic differentiation and to increase the potential of self-organizing organoid formation.

In this study, we aim at investigating the contribution of extrinsic signals secreted by the cells, in response to developmental exogenous signals, to the progression from pluripotency to the hepatic lineage and their competence to generate nascent hepatic organoids. To achieve this aim, we envision performing hepatic differentiation in the microfluidic confined environment where, thanks to the low volume to cellular surface ratio, factors secreted by the cells are rapidly accumulated.

We previously demonstrated that the controlled balance between soluble endogenous factors versus exogenous factors in microfluidics (μF) has an impact on pluripotency maintenance, germ layer specification, and hepatic differentiation of hPSCs (Giobbe et al., 2015). In particular, we found that endoderm commitment and hepatic differentiation were affected by the frequency of media change in μF, suggesting the efficiency of differentiation is correlated with the accumulation of endogenous factors. Similarly, we demonstrated that increased efficiency in reprogramming of adult somatic cells into hiPSCs in μF can be ascribed to the accumulation of endogenous cell-secreted factors (Luni et al., 2016; Giulitti et al., 2019).

Here, we show that the confined environment in μF emphasizes the response of cells to extrinsic secreted factors, with implications on the phenotype and functional differentiation of hepatocyte-like cells. We characterized the cellular secretome during early stages of differentiation by high-throughput proteomic analysis for a quantitative comparison of protein abundance between μF and conventional culture conditions (CCC). We found that protein accumulation (in particular ECM-related proteins) in μF is two orders of magnitude higher compared to CCC. This ECM-enriched secretome significantly enhances the potential of immature hepatocytes to form 3D hepatic organoids and their further differentiation to mature hepatocytes. Similarly, the exogenous supplementation of core ECM components in CCC during the early stage of hepatic differentiation allows us to generate hepatic organoids with higher functional activities. These findings provide insights into the role of the secretome during human liver organogenesis and for efficiently and robustly deriving hepatic organoids from hiPSCs.

## Results

### The Confined Environment Boosts Hepatic Differentiation

We first developed a robust and effective protocol for the hepatic differentiation of hPSCs toward definitive endoderm (DE), hepatic endoderm (HE), immature hepatocytes (IHs), and functional hepatocytes (MHs) in μF.

We found that when hPSCs are seeded at high density in μF in endoderm differentiation medium, the exit from pluripotency is restricted to few *FOXA2*^+^ cells that segregate from *OCT4*^+^ cells (Figure S1A). On the other hand, low density-seeded hPSCs in μF allows obtaining *FOXA2*^+^ cells with a minor subpopulation of *OCT4*^+^ cells after 3 days of endoderm induction (Figure S1A). This result is consistent with our previous observation in which a high frequency of intermittent medium change (8 times per day) promotes endoderm commitment of hPSCs in μF, thanks to a sustained wash-out of endogenous cell-secreted factors (Giobbe et al., 2015). Conversely, when accumulation of endogenous factors is promoted, we obtained hepatocyte-like cells with higher Albumin secretion and cytochrome activity in a shorter period of time in μF compared to CCC (Giobbe et al., 2015).

**Figure 1:**
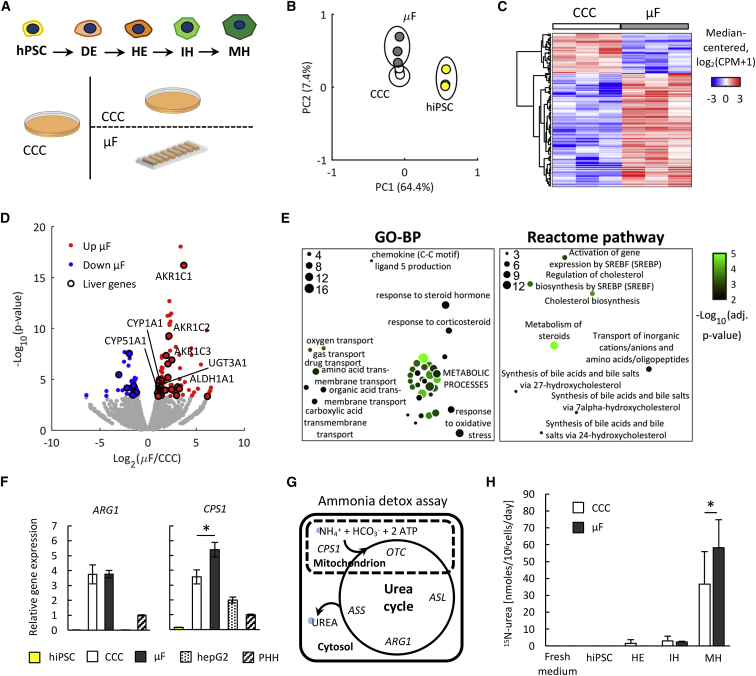
Characterization of MH Cells Derived through the Two-Phase Differentiation Protocol (A) Outline of the 2-phase hepatic differentiation protocol of hPSCs to DE, HE, IH, and MH. In the initial phase, DE cells are derived in CCC, split, injected into microfluidic channels for the second phase, and cultured with a low frequency of intermittent medium change (2 times per day). DE cells were also re-plated with the same split ratio and differentiated in CCC as control. (B) PCA of MH cells derived from H0-193 hiPSCs in μF or in CCC, obtained from RNA sequencing (RNA-seq) data. (C) Hierarchical clustering shows differentially expressed genes (DEGs) between MH cells derived in μF and in CCC. (D) Volcano plot highlights DEGs (fold change [FC] >1.5, false discovery rate [FDR] <0.05) and, among them, liver-specific genes (black outline). Hepatocyte-specific enzymes upregulated in μF are highlighted. (E) Functional enrichment analysis within Gene Ontology-biological process (GO-BP) and Reactome pathway categories of DEGs upregulated in μF highlights enrichment of metabolic pathways. Dot size is proportional to the number of genes and green intensity to the p value, according to the legend. (F) Real-time PCR analysis of urea-cycle genes *ARG1* and *CPS1* of MH cells derived from H0-193 hiPSCs in μF or in CCC. Undifferentiated hiPSCs were used as negative control. Immortalized hepatic cell line HepG2 and primary human hepatocytes (PHH) were used as positive controls. Mean ± SE, n = 4, t test, ^∗^p value <0.01. (G) Ammonia detoxification assay through administration of heavy-labeled ammonium chloride and measurement of secreted heavy-labeled urea. (H) Quantification of labeled urea in supernatants after 48 h heavy ammonium chloride administration to undifferentiated, HE, IH, and MH cells derived in μF and in CCC. Mean ± SE, n = 4, t test, ^∗^p value <0.01.

With these results, DE cells derived in CCC (phase 1) are seeded in μF and differentiated to HE, IHs, and MHs (phase 2) (Figure 1A). We tested this strategy by adapting different hepatic differentiation protocols reported in the literature (Hay et al., 2008; Cai, 2014) (Figure S1B). We obtained hepatocyte-like cells with homogeneous expression of adult hepatic markers *HNF4*α, *ALB*, *AAT*, *CYP1A2*, and *CYP3A4*, evidence of bile canaliculi-like structures and *MRP2* expression (Figure S1C), along the microfluidic channels. Consistent results were obtained with different hiPSC lines (Figure S1D), confirming the robustness of this two-phase differentiation strategy. “Protocol #2” in Figure S1B will be used throughout the manuscript, because it is based on serum-free and chemically defined media, which represents a major requirement to perform secretome analysis.

We then asked whether the confined environment is effectively supporting the hepatic differentiation of hPSCs by providing a transcriptomic signature of MH cells derived in μF compared to those derived in CCC, according to the experimental set up reported in Figure 1A. Principal component analysis (PCA) shows separated clusters of pluripotent and differentiated cells and a smaller, but well-defined, separation between MH cells in μF and CCC (Figure 1B). A number of genes were found to be differentially expressed, as shown in Figure 1C, where hierarchical clustering indicates 115 genes upregulated in μF and 40 genes downregulated (Data S1). 37 of all differentially expressed genes (DEGs) are known to be expressed in the adult hepatic tissue and, interestingly, 30 of these liver genes (81%) were upregulated in μF (Figure 1D). Among the genes upregulated in μF, we identified liver cytochromes (*CYP1A1* and *CYP51A1*), aldoketoreductases (*AKR1C1*, *AKR1C2*, and *AKR1C3*), aldehyde dehydrogenases (*ALDH1A1*), and uridine diphosphate (UDP) glycosyltransferases (*UGT3A1*), albeit some of them are associated to a fetal stage of development. We further investigated the functional significance of the genes upregulated in μF by performing an enrichment analysis. Figure 1E shows enrichment of multiple categories related to metabolic processes, including typical hepatic functions such as “synthesis of bile acids and bile salts” and “cholesterol biosynthesis,” suggesting higher differentiation of MH cells in μF (Camp et al., 2017). Overall, this suggests that the confined environment promotes the expression of genes associated with hepatic metabolism.

We further analyzed the functional activity of MH cells in terms of ammonia detoxification through urea production, a key liver metabolic function (Yu et al., 2012b). Indeed, hepatocytes only are able to metabolize ammonia through the complete urea cycle, a sequence of enzymatic and transport steps necessary to metabolize and excrete the nitrogen generated by the breakdown of amino acids in protein and other nitrogen-containing molecules. We first assessed the expression of key urea cycle genes (*ARG1*, *ASL1*, *ASS1*, *CPS1*, and *OTC*) using primary human hepatocytes, previously tested for their functional activity (Figure S2A), as positive control. Remarkably, we observed a significant 1.5-fold increase of the rate-limiting mitochondrial enzyme *CPS1* expression in μF compared to CCC (Figures 1F and S2B).

In order to functionally validate our findings, we also developed a procedure to measure the urea released in cell culture supernatants that measures both the urea derived from ammonia detoxification and the urea derived from other sources (e.g., Arginine). We exposed MH cells to ammonium chloride labeled with heavy nitrogen (^15^N), in order to track ammonia nitrogen through the urea cycle to urea with the molecular weight of urea +1 (Figure 1G). To allow measurement of unlabeled urea, ^15^N-urea and the internal standard ^13^C,^15^N_2_-urea in microfluidic samples, a gas-chromatography mass spectrometry assay was specifically developed (Figures S2C and S2D). Figure 1H shows a significant increase of labeled urea released from MH cells in μF compared to CCC, confirming increased urea cycle function, whereas, as expected, no significant differences were observed for HE and IH cells. These results demonstrate that in addition to a different transcriptomic signature between μF and CCC, confined environment promotes increased ammonia detoxification in μF.

### Stable Isotope Labeling by Amino Acids in Cell Culture (SILAC) Analysis Reveals Accumulation Of Self-Produced ECM Proteins in μF

We hypothesized that the accumulation of endogenous cell-secreted factors in μF promotes hepatic commitment of DE cells to the hepatic fate. As a matter of fact, we observed phenotypic differences among μF and CCC as early as the transition from HE to IH cells (Figure S3A) between days 10 and 15. In particular, we observed a more defined epithelial phenotype, characterized by a clear polygonal cytoskeletal *F-ACTIN* arrangement of *AFP*^+^ cells (Figure S3B).

In order to identify the endogenous cell-secreted factors accumulated in μF, we designed a comprehensive proteomic study of cell-secreted proteins in conditioned media, using liquid chromatography-tandem mass spectrometry (LC-MS/MS) after SILAC labeling according to our previously described methodology (Hu et al., 2018b). This has been done in order to quantitatively compare the cell secretome in μF and CCC, which requires different labeling of cell-secreted proteins. Specifically, we labeled cells in μF and CCC with heavy and light amino acids, respectively. Then, we mixed 1:1 volume-based conditioned media collected in the two culture systems in order to analyze the ratio of concentration of each protein identified by LC-MS/MS analysis. Moreover, labeling with heavy amino acids ensures discrimination between cell-secreted proteins and those already present in culture media.

**Figure 2:**
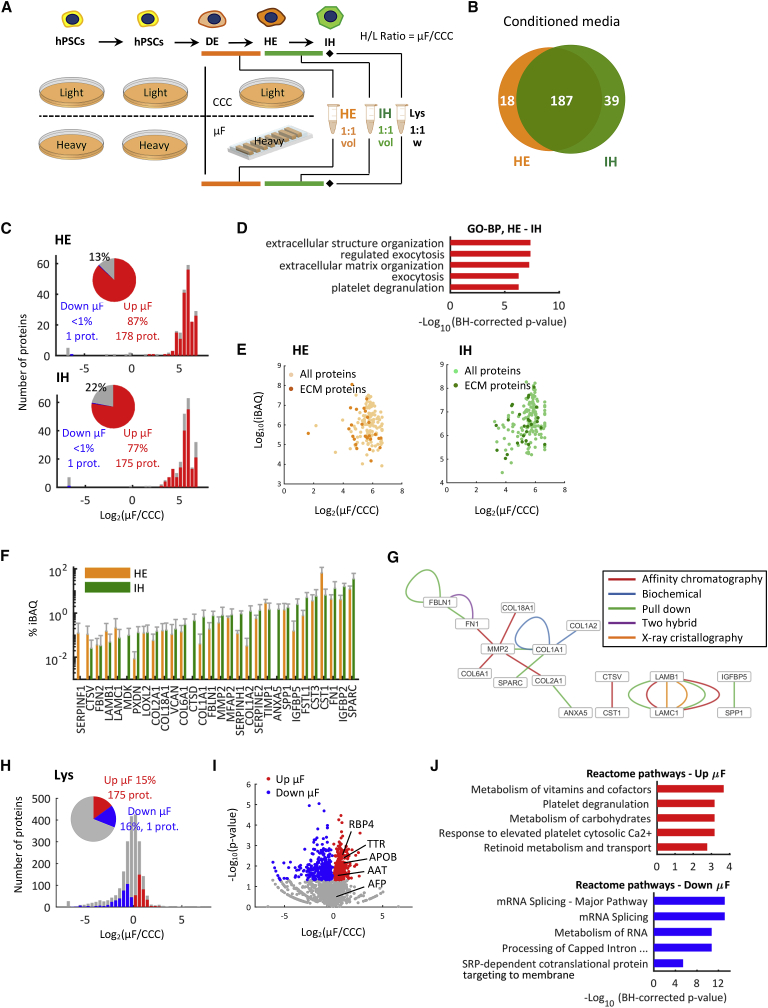
SILAC Proteomic Analysis during Early Stage Hepatic Differentiation (A) Experimental set up of SILAC proteomic analysis. hPSCs were labeled with light and heavy amino acids and differentiated to IH cells in CCC (light-labeled cells) and μF (heavy-labeled cells) to analyze heavy/light proteins ratios (H/L ratio) in conditioned media and lysates. (B) Venn diagram of detected proteins in conditioned media of HE and IH cells derived from H0-193 hiPSCs and collected according to the experimental set up in (A). (C) Histograms of secreted proteins detected in HE and IH samples, according to their H/L ratio. Proteins significantly accumulated in μF and in CCC are indicated in red and blue, respectively. The remaining proteins are shown in gray. Insets: pie plots of the percentage of proteins in these three categories. (D) Functional enrichment analysis within GO-BP categories of proteins significantly accumulated in μF in HE and IH samples reveals extracellular matrix-related categories on top. (E) iBAQ values versus SILAC ratios of proteins significantly accumulated in μF in HE and IH samples. ECM-related proteins are highlighted in dark orange and dark green in HE and IH samples, respectively. (F) Bar plot of iBAQ values of ECM-related proteins significantly accumulated in μF. iBAQ is expressed as percentage of the total amount of ECM-related proteins in HE and IH samples, respectively. (G) Network of the protein-protein interactions derived by previous experimental studies using the techniques indicated in the legend. All ECM-related proteins upregulated in μF were included in the analysis, but only proteins with at least one known interaction are reported. (H) Histogram of proteins identified in IH cells lysates, according to their SILAC ratio. Proteins significantly up- and downregulated in μF are indicated in red and blue, respectively. The remaining proteins are shown in gray. Insets: pie plots of the percentage of proteins in these three categories. (I) Volcano plot of the same proteins shown in (H). Hepatic markers *TTR*, *RBP4*, *APOB*, and *AAT*, but not *AFP*, are overexpressed in μF. (J) Functional enrichment analysis of Reactome pathways of proteins up- and downregulated in μF highlights metabolic pathways and DNA transcription as top-ranking categories, respectively.

We first adapted the two-phase hepatic differentiation protocol in order to optimize LC-MS/MS measurement coupled with SILAC conditions, which requires media with low protein content and no sources of exogenous un-labeled amino acids (STAR Methods). We expanded hPSCs to allow the complete incorporation of heavy and light amino acids, differentiated them into IH cells and collected conditioned media from DE to HE and from HE to IH stages, in μF and CCC (Figure 2A). Cell lysates were also collected at the end of the experiment and mixed 1:1 weight-based in order to analyze the intracellular content. No alterations of the hepatic differentiation outcome have been observed using SILAC-compatible media (data not shown).

We quantified 205 and 226 proteins in the HE and IH samples, respectively, with 77% of proteins in common between the two stages (Figure 2B). Correlation among replicates was verified through the Pearson’s correlation coefficient (Figure S3C). The majority of the identified proteins resulted in accumulation in μF for both HE and IH with a μF/CCC ratio >1 (Figure 2C). Similar results were obtained with an hESC line (Figures S3C–S3E). We therefore investigated the functional role of the cell-secreted proteins accumulated in μF. Approximately 74% of these proteins are exosome proteins (GO-CC:0070062) and 22% extracellular matrix proteins (GO-CC:0031012) (Data S2). Remarkably, the top ranking category of Gene Ontology-biological process (GO-BP) enrichment analysis is “extracellular structure organization” (GO-BP:0043062) for both hiPSC and hESC lines (Figures 2D and S3F). Core ECM proteins significantly accumulated in μF included 11 ECM glycoproteins (such as *SPARC*, *FN*, *LAMB1*, and *LAMC1*), 5 collagens (*COL1A1*, *COL1A2*, *COL2A1*, *COL6A1*, and *COL18A1*), and 1 proteoglycan (*VCAN*). Besides structural ECM components, we also identified 13 ECM-associated proteins, including proteases (*MMP2* and *CTSV*) and protease inhibitors (*CST1/3*, *TIMP1*, *SERPINF1*, and *SERPINH1*). The abundance of these ECM-related proteins overall accumulated with ratios up to 7 in μF spans almost four orders of magnitude in terms of intensity-based absolute quantification (iBAQ) values, which are proportional to the molar quantities of the proteins (Figure 2E). Interestingly, the most abundant protein is *SPARC* (Figure 2F), a glycoprotein involved in the regulation of cell shape, adhesion, migration, and proliferation, playing a major role in cell-matrix interactions and collagen binding (Daley et al., 2008). A protein-protein interaction network analysis revealed known experimentally validated physical interactions among many of these proteins (Figure 2G), which are likely part of a network of activation and repression of extracellular signals mediated by enzyme activity partnered with ECM sequestration and release of signals (Bonnans et al., 2014). Collectively, these results provide evidence for accumulation of soluble ECM-related proteins, functionally related among each other, during early stages of hepatic differentiation.

In order to confirm that secretion and accumulation of these ECM-related proteins in μF is not due to an increased synthesis of the same proteins, we analyzed cell lysates at the end of IH stage in μF and CCC (Figure 2H). A total of 2,234 proteins were identified in at least two replicates, and 30% of them (685 proteins) were differentially expressed among μF and CCC. Among these, 48% were significantly overexpressed in μF and 52% in CCC (Figure 2I). Remarkably, 90% of the 30 ECM-related proteins that significantly accumulated in μF conditioned media were not overexpressed in μF lysates (μF/CCC ratio <1), thus confirming they are accumulated and not over-translated. Conversely, hepatic markers such as *TTR*, *RBP4*, *APOB*, and *AAT*, but not *AFP*, were found to be overexpressed in μF (Figure 2I), as further confirmed by immunostaining (Figures S3G and S3H) and qPCR analysis (Figure S3I).

We investigated GO categories related to the upregulated proteins in μF, which mostly included metabolic pathways as top-ranking GO-BP categories. Of note, categories with less statistical significance included, among others, well-known hepatic specific function-related pathways “gluconeogenesis,” ”glycolysis,” regulation of cholesterol biosynthesis,” and ”glucose metabolism” (Data S3), confirming a more differentiated phenotype of IH cells in μF. Conversely, DNA transcription-related categories were found at the top list of proteins overexpressed in CCC (μF/CCC ratio <1) GO enrichment analysis (Figure 2J).

Overall, these data demonstrate that the ECM-related proteins previously identified derive only from the accumulation induced by the confined environment and not from intracellular synthesis. Moreover, GO analysis confirms IH cells in μF display a more differentiated phenotype (Yang et al., 2017b), compared to CCC.

### Accumulation of Soluble ECM Proteins Results in Increased Deposition and Remodeling

**Figure 3:**
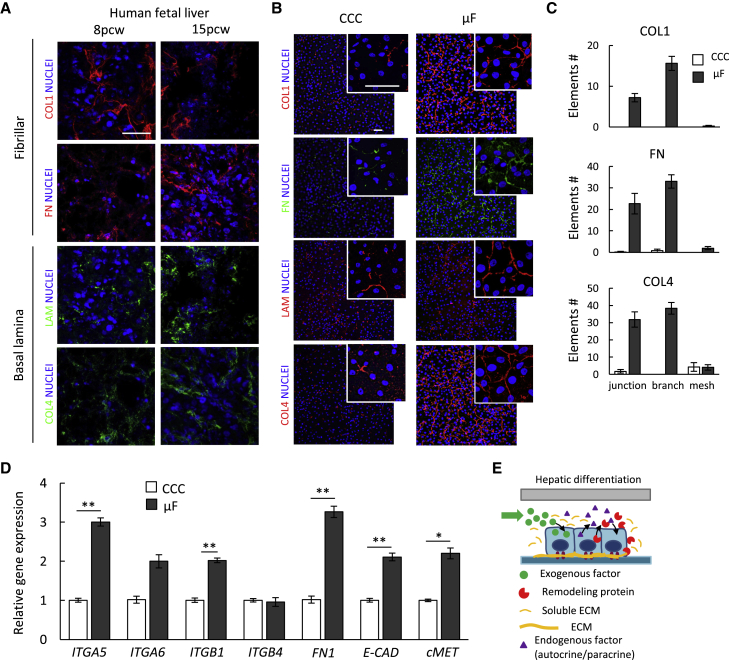
ECM Remodeling Analysis in μF (A) ECM proteins in human fetal liver tissue slices obtained from 8 and 15 pcw human embryos. *COL1*, *FN*, *LAM*, and *COL4* are all expressed at both fetal stages. Scale bar, 10 μm. (B) *COL1*, *FN*, *LAM*, and *COL4* expression of IH cells derived from H0-193 hiPSCs in CCC and μF. Scale bar, 10 μm. (C) Quantification of the net-like structure by means of image processing analysis through the number of junctions, branches, and meshes in μF compared to CCC. Mean ± SE, n = 6. (D) Real-time PCR analysis of Integrin receptors (*ITGA5*, *ITGA6*, *ITGB1*, and *ITGB4*), *FN1*, epithelial markers (*E-CAD* and *cMET*) of IH cells derived from H0-193 hiPSCs in CCC and μF. Mean ± SE, n = 6, t test, ^∗^p value <0.01, ^∗∗^p value <0.005. (E) Proposed model of hepatic differentiation driven by exogenous factors, endogenous factors, and endogenously produced ECM-related proteins, including remodeling enzymes and structural components, which collectively give rise to deposition and remodeling of a “hepatic-specific” insoluble ECM matrix.

Given the enhanced accumulation of ECM-related proteins in μF, we used immunofluorescence analysis to investigate the deposition of structural basal lamina components *COL4* and *LAM* and fibrillar proteins *COL1* and *FN*. We first evaluated the expression of these proteins in the human fetal liver at different developmental stages (i.e., 8 and 15 post-conception weeks, pcw) (Figure 3A), confirming that they have a role in human liver development. We then performed the same analysis on hiPSC-derived IH cells in μF and CCC. Figure 3B shows a substantially higher and more widespread expression for all the analyzed proteins in μF compared to CCC. Moreover, we observed a well-defined net-like structure, potentially arising from an amplified remodeling activity in μF. We quantified the net-like structure for *COL4*, *COL1*, and *FN* by means of image processing analysis (STAR Methods), identifying a higher number of junctions, branches, and meshes in binary converted images in μF compared to CCC (Figures 3C and S4A). We excluded *LAM* from this quantification to avoid any bias due to the elevated signal from the laminin contained in the Matrigel coating. Yet a significant remodeling can be qualitatively appreciated.

We next investigated if the increased deposition of ECM proteins in μF is associated with overexpression of ECM-receptors in IH cells derived in μF compared to CCC. qPCR analysis revealed that a set of integrins involved in the binding to fibronectin and collagens (i.e., *ITGA5*, *ITGA6*, and *ITGB1*) are overexpressed in μF (Figure 3D). Interestingly, ECM deposition and remodeling in μF is also associated with a significant upregulation of the *FN1* gene, epithelial markers *E-CAD*, and *c-MET*, which are widely recognized to be involved in hepatic differentiation by binding the exogenous HGF.

We concluded that cell-secreted endogenous factors accumulated in the confined environment include structural ECM proteins, protease, and proteases inhibitors (Figure 3E), which collectively contribute to produce and remodel a proper extracellular “niche” for the progression from pluripotency to the hepatic lineage. Strikingly, this is associated with changes in the cell transcriptome that involve the overexpression of cell-ECM receptors.

### Exogenous ECM Supplementation Enhances the Formation of Functional Hepatic Organoids

**Figure 4:**
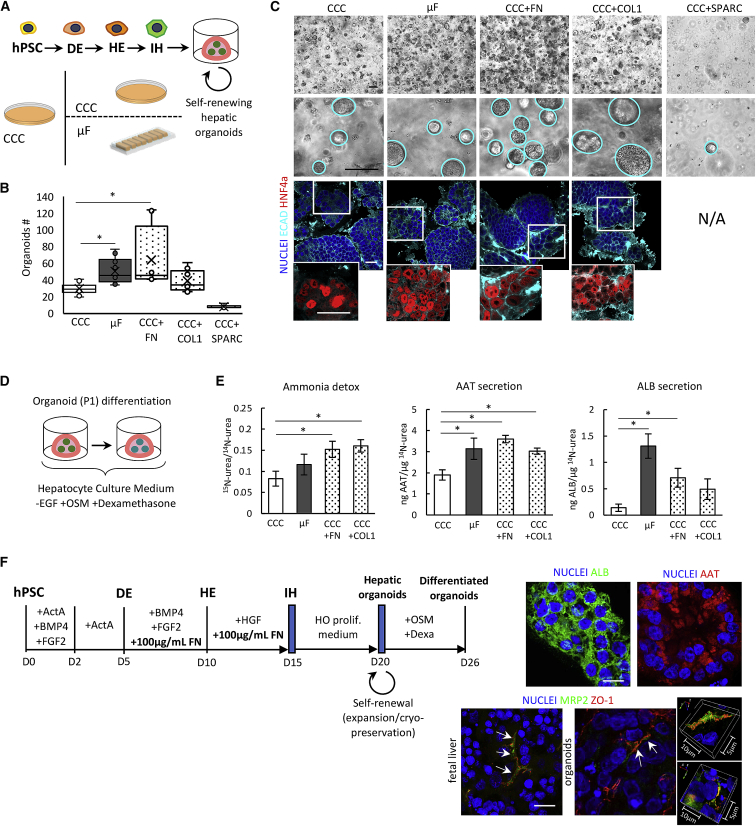
Hepatic Organoid Formation and Differentiation (A) Experimental set up of hepatic organoids formation of ECM-treated cells. IH cells were derived from H0-193 hiPSCs in CCC with the exogenous supplementation of 100 μg/mL rat tail *COL1*, 100 μg/mL bovine *FN*, or 10 μg/mL of recombinant *SPARC* from DE to IH stage, dissociated at single cell and embedded in 3D Matrigel drops in self-renewing hepatic organoid medium. (B) Boxplot representing the number of organoids recognized after 6 days from single cells embedding for different conditions. n = 6–10, one-way ANOVA, ^∗^p value <0.05. (C) Top: organoids formation of ECM-treated cells from H0-193 hiPSCs were compared with cells derived in CCC or in μF with no treatment. Scale bar, 50 μm. Bottom: immunostaining analysis shows homogeneous expression of epithelial *E-CAD*, and hepatic *HNF4α* markers in all the conditions tested. Scale bar, 50 μm. (D) Outline of self-renewing hepatic organoids differentiation through supplementation of OSM and dexamethasone for 6 days. (E) Functional assay of differentiated organoids. Quantification of labeled urea in supernatants after 24 h heavy ammonium chloride administration to differentiated organoids. ELISA assays for detecting *AAT* and *ALB* secreted in 24 h from differentiated organoids. Mean ± SE, n = 6, one-way ANOVA, ^∗^p value <0.05. (F) Protocol for the derivation and differentiation of hepatic organoids from hiPSCs including the addition of soluble FN (left). Immunostaining of differentiated organoids shows homogeneous expression of *ALB* and *AAT* markers and evidence of hepatocyte polarity similar to fetal hepatic tissue (right). Scale bar, 10 μm.

The upregulation of ECM receptors, and particularly integrins, is recognized to be key for the formation of 3D organoids, particularly of endodermal origin (Olabi et al., 2018; Hernandez-Gordillo et al., 2019). Therefore, we tested the potential of our hiPSC-derived IH cells exposed to the self-organizing ECM protein network in μF to generate hepatic organoids (Figure 4A), by harnessing a self-renewing medium recently optimized for hepatic progenitor cells directly isolated from fetal or adult tissue (Hu et al., 2018a).

Strikingly, we observed a significantly higher number of nascent organoids from IH cells derived in μF compared to CCC (Figure 4B). We also asked whether the exogenous supplementation of soluble ECM proteins in CCC—from DE to IH cells and before organoids formation—allows for a similar improvement. We selected the two most abundant core ECM proteins identified through the SILAC analysis (i.e., *FN* and *COL1*), as well as *SPARC*, a collagen-binding ECM glycoprotein, ranking at top of the proteomic analysis (Figure 2F). We then tested if a “soluble ECM-treatment” in CCC was sufficient to obtain IH cells competent to organoid formation similarly to IH cells obtained in μF. ECM-treated IH cells appeared all morphologically different compared to both control CCC and μF (Figure S4B), with increased *COL1* deposition in *COL1*-treated cells compared to both CCC and other ECM treatments. Again, substantial differences in the number of nascent organoids emerged compared to CCC without treatment. Specifically, we observed a significant increase in the number of organoids from both *COL1*- and *FN*-treated cells, compared to non-treated cells (CCC). In particular, *FN*-treatment (CCC+FN) resulted in a 2-fold increase in the number of nascent organoids, whereas only a slight but not significant increase has been observed following *COL1*-treatment. Conversely, *SPARC*-treated cells were not competent for giving rise to hepatic organoids, resulting in a severe decrease compared to cells derived in CCC (Figure 4B) and therefore excluded for further experiments. Because *SPARC* is recognized to be a remodeling protein with a key role in collagen binding and metalloproteinase activity (Barker et al., 2005), we speculate *SPARC* has a role in combination with other ECM core components, rather than alone. No significant differences in organoids dimensions have been observed among the different conditions (Figure S4C).

On day 6 after 3D embedding, nascent hepatic organoids can be clearly recognized with a well-defined round shape while expressing epithelial *E-CAD* and *HNF4α* in all the conditions tested (Figure 4C). Hepatic organoids, including those derived with exogenous supplementation of *FN* and *COL1*, can be expanded for at least 4 passages, while maintaining self-renewing properties and round-shape morphology and cryopreserved for later usage (data not shown). We also tested the ability of these organoids as early as passage 1 to differentiate into functional hepatocytes through the supplementation of OSM and dexamethasone (Figure 4D). After 6 days of differentiation, we overall observed a significant increase in ammonia detoxification, α1-antitrypsin (*AAT*), and albumin (*ALB*) secretion in organoids derived from μF, *COL1*-, and *FN*-treated IH cells, compared to the negative control (i.e., CCC) (Figure 4E).

These results confirm that IH cells derived in a highly remodeled extracellular environment arising from ECM proteins accumulation display an enhanced potential to form hepatic organoids that can be further differentiated to functional hepatocytes. Moreover, the exogenous supplementation of soluble ECM proteins could compensate the accumulation of native proteins in μF. In particular, *FN*-treatment resulted in the most efficient in terms of both organoid formation and further differentiation. This opens up the possibility of developing a protocol of human liver organogenesis where the supplementation of additional soluble ECM supports the generation, and functional differentiation of hepatic organoids form hiPSCs within 26 days (Figure 4F). Immunostaining analysis of organoids derived with this protocol display homogeneous expression of *AAT* and *ALB*, as well as evidence of polarity with confined colocalization of *ZO-1* and *MRP2* markers (Figure 4F), similar to a liver tissue. Overall, these results provide a robust tool to derive hepatic organoids from hiPSCs for expansion purposes or for disease modeling applications, potentially overcoming donor-to-donor variability.

## Discussion

In this work, we analyzed the whole secretome of endoderm-committed hPSCs during hepatic differentiation, using the microfluidic technology as a tool to enhance the accumulation of cell-secreted factors. This allowed us to identify a key role of ECM and ECM remodeling proteins in the extrinsic regulation of hepatic differentiation, relevant to the study of human liver development. We applied a SILAC-MS-based quantitative proteomic analysis to fully characterize the extracellular environment promoting the progression toward the hepatic lineage. SILAC labeling has a double advantage for secretome analysis. First, it allows for a very accurate relative quantification of proteins in the conditioned media from the two culture systems (i.e., μF and CCC). Second, it ensures the proteins labeled as heavy (in μF) are secreted from the cells and not derived from media impurities or other protein contaminations (Ong et al., 2002). It is important to notice that, even if specifically developed for μF, this adapted protocol does not require cell starvation before the analysis or other protocol modifications that would reduce the significance of the biological information for broader applicability. Moreover, based on our previous study, we exclude any significant alterations in terms of protein absorption on *polydimethylsiloxane* (PDMS), nutrients, or oxygen depletion due to the microfluidic culture (Hu et al., 2018b; Giobbe et al., 2015).

Specifically, we succeeded in measuring: (1) the accumulation of endogenously produced cell-secreted proteins in supernatants collected in μF compared to those collected in CCC, and (2) protein expression differences between μF and CCC. Among cell-secreted proteins accumulated in μF, ECM-related proteins, including structural ECM components such as *FN*, fibrillar collagens, and laminins (but also a set of proteases and protease inhibitors that likely contribute to their remodeling) constituted around 15% of all the identified proteins and clustered at the top of GO-enrichment analysis. Remarkably, an experimentally validated physical interaction network between these proteins confirmed their functional connection.

Moreover, with a defined net-like structure, we identified a substantial higher and more widespread deposition of these ECM proteins in μF compared to CCC. Whereas both *LAM* and *COL4* are highly abundant in the MRF-coating solution that we used to functionalize cell culture substrates, and therefore already present, both *COL1* and *FN* likely arise almost entirely from the deposition of soluble self-produced ECM. Therefore, we demonstrated the confined environment not only promotes the accumulation of cell-secreted proteins but also their functional activity of proteolytic cleavage of ECM components and remodeling (Daley et al., 2008). We speculate that this remodeling likely induces recruitment and activation of transmembrane proteins, such as integrins, which cluster on the cell membrane forming focal adhesions and trigger specific signaling pathways, impacting cell differentiation. In fact we observed overexpression of integrins, particularly *ITGA5* and *ITGB1*, that are specifically involved in the cell binding with ECM proteins. Moreover, consistent with previous results, *FN*-mediated integrin overexpression in hepatic cells seeded on *FN*- and *COL1*-functionalized scaffolds (Wang et al., 2016b). We also found that higher expression levels of *c-MET* are likely to be associated with integrins that have been shown to activate *c-MET* in an HGF-independent manner (Mitra et al., 2011). Of note, the implication of a specific ECM has been recognized as an important modulator of liver organogenesis (Ober and Lemaigre, 2018; Handa et al., 2014).

We also demonstrated that the accumulation of cell-secreted proteins leads to a different signature of MH cells in μF compared to those derived in CCC, with a significant upregulation of hepatic metabolic pathways (Godoy et al., 2015), suggesting a more mature phenotype in μF and consistent with our previous results (Giobbe et al., 2015). Remarkably, we also reported ammonia detoxification through the urea cycle, a key function of hepatocytes that is not present in many hepatic cell lines, which can generate urea but not detoxify ammonia (Mavri-Damelin et al., 2007).

Native ECM secretion and deposition are likely to play a more relevant role in a 3D context (Loebel et al., 2019), which is closer to the physiological *in vivo* environment of the hepatic tissue as well as indispensable for the establishment of the proper hepatocytes polarity (Zeigerer et al., 2017). In this perspective, the key contribution of native ECM and upregulation of specific integrin receptors involved in the cell-ECM binding during differentiation can be effectively appreciated in the 3D hepatic organoids generation and differentiation. We demonstrated that the exposure of hepatic progenitors to a soluble microenvironment enriched in endogenously produced ECM allows for enhanced hepatic organoid formation compared to control cells differentiated in CCC. Self-renewal of these hepatic organoids was maintained for several passages with culture conditions optimized for long-term expansion of human primary hepatocytes (Hu et al., 2018a). Moreover, self-renewing hepatic organoids can be differentiated toward functional hepatocytes, in terms of ammonia detoxification, *AAT*, and *ALB* secretion, compared to those derived from cells differentiated in CCC.

3D hepatic organoids have been recently developed to recapitulate *in vitro* stages of human liver organogenesis from hiPSCs, ultimately leading to more mature and functional hepatocytes compared to 2D culture systems. Indeed, hepatic organoids have the potential to fairly replicate key aspects of human liver tissue, in particular its complex architecture and metabolic functions, as well as to recapitulate the pathogenesis of metabolic diseases. Different protocols for the generation of self-renewing hepatic organoids from hiPSCs have been proposed, for instance, based on the spontaneous generation of 3D spheroids from endodermal cells (Guan et al., 2017; Akbari et al., 2019). More complex human liver bud models also allowed for a dissection of the crosstalk between parenchymal and non-parenchymal cells inducing liver development (Takebe et al., 2013; Asai et al., 2017; Camp et al., 2017), confirming a major role for paracrine signals from mesenchyme in specifying cells to the hepatic fate.

Compared to these protocols, we demonstrated that the contribution of extrinsic signaling arising from soluble cell-secreted ECM accumulation is key to promote the formation of hepatic organoids, as well as their functional differentiation. Interestingly, the supplementation of soluble *FN* during differentiation, allows for obtaining IH cells highly competent for organoids generation, similarly to IH cells derived in μF. Therefore, we propose a rapid and efficient method to derive self-renewing hepatic organoids from hiPSCs, with the potential to be differentiated into hepatocytes with some functional activities within 25 days (Figure 4F).

In conclusion, we reported that μF coupled with SILAC-MS-based quantitative proteomic analysis allowed us to investigate the extrinsic regulatory network of cell-secreted factors, which are likely to have a major role in shaping the extracellular microenvironment and, consequently, affect stem cell differentiation (Loebel et al., 2019; Qiao et al., 2019). These findings provide further insights into hiPSC-based models of human liver organogenesis using the organoid technology, with a major impact on disease modeling and regenerative medicine applications (Schwartz et al., 2012; Yang et al., 2017a; Warren et al., 2017).

## STAR★Methods

### Key Resources Table

**Table:** 

REAGENT or RESOURCE	SOURCE	IDENTIFIER
**Antibodies**		

Mouse monoclonal anti-α-Fetoprotein (AFP)	Sigma-Aldrich	Cat# A8452; RRID: AB_258392
Rabbit polyclonal anti-HNF-4α	Santa Cruz Biotechnology	Cat# sc-8987; RRID: AB_2116913
Goat polyclonal anti-Human α1-Antitrypsin (AAT)	R&D Systems	Cat# AF1268; RRID: AB_354707
Mouse monoclonal anti-Human Serum Albumin (ALB)	R&D Systems	Cat# MAB1455; RRID: AB_2225797
Rabbit polyclonal anti-ZO-1	Genetex	Cat# GTX108627; RRID: AB_10731582
Mouse monoclonal anti-CYP1A2	Genetex	Cat# GTX84643; RRID: AB_10727429
Rabbit polyclonal anti-CYP3A4	Genetex	Cat# GTX117120; RRID: AB_10617497
Mouse monoclonal anti-MRP2	Abcam	Cat# ab3373; RRID: AB_303751
Rabbit polyclonal anti-Collagen I	Abcam	Cat# ab34710; RRID: AB_731684
Rabbit polyclonal anti-Collagen IV	Abcam	Cat# ab6586; RRID: AB_305584
Rabbit polyclonal anti-Laminin	Sigma-Aldrich	Cat# L9393; RRID: AB_477163
Mouse monoclonal anti-Fibronectin	Sigma-Aldrich	Cat# F7387; RRID: AB_476988

**Chemicals, Peptides, and Recombinant Proteins**		

Recombinant Human Activin A	R&D Systems	Cat# 338-AC
Recombinant Human FGF-basic	Peprotech	Cat# 100-18B
Recombinant Human BMP4	Peprotech	Cat# 120-05
Recombinant Human HGF	Peprotech	Cat# 100-39H
Recombinant Human Oncostatin M	R&D Systems	Cat# 295-OM
Dexamethasone	Sigma-Aldrich	Cat# D4902; CAS: 50-02-2
Collagen I, rat tail	BD Biosciences	Cat# 354236
Fibronectin bovine plasma	Sigma-Aldrich	Cat# F1141
Recombinant SPARC	Generon	Cat# CSB-RP094444h

**Deposited Data**		

RNA-seq data	This paper	GEO: GSE159926
Proteomic data	This paper	Massive: MSV000084128

**Oligonucleotides**		

Taqman probes for quantitative real-time PCR, see Table S1	Thermo Fisher Scientific	N/A

**Software and Algorithms**		

bcl2fastq v2.20.0.422	Illumina proprietary software	https://emea.support.illumina.com/sequencing/sequencing_software/bcl2fastq-conversion-software.html
Bbduk (bbmap suite 37.31)	Joint Genome Institute	https://jgi.doe.gov/data-and-tools/bbtools/
STAR 2.6.0a	Dobin et al., 2013	https://github.com/alexdobin/STAR
R version 3.5	R Foundation for Statistical Computing (2017)	https://www.R-project.org
EdgeR v. 3.5.1	Robinson et al., 2010	Bioconductor package (https://www.bioconductor.org/)
ReactomePA v. 1.28.0	Yu and He, 2016	Bioconductor package (https://www.bioconductor.org/)
ClusterProfiler v. 3.12.0	Yu et al., 2012a	Bioconductor package (https://www.bioconductor.org/)
MATLAB R2017a	Commercial software	https://www.mathworks.com/products/matlab.html
Thermo Proteome Discoverer v. 2.2	Commercial software	https://www.thermofisher.com/us/en/home/industrial/mass-spectrometry/liquid-chromatography-mass-spectrometry-lc-ms/lc-ms-software/multi-omics-data-analysis/proteome-discoverer-software.html
MaxQuant v.1.6.2	Cox and Mann, 2008	https://www.maxquant.org/
Cytoscape v.3.7	Shannon et al., 2003	https://cytoscape.org

**Others**		

RNA-sequencing data analysis, see Data S1	This paper	N/A
Secretome proteomic data analysis, see Data S2	This paper	N/A
Lysate proteomic data analysis, see Data S3	This paper	N/A

### Resource Availability

#### Lead Contact

Further information and requests for resources and reagents should be directed to and will be fulfilled by the Lead contact, Nicola Elvassore (nicola.elvassore@unipd.it).

#### Materials Availability

This study did not generate new unique reagents.

#### Data and Code Availability

The LC-MS/MS proteomics data generated during this study have been deposited to the Mass Spectrometry Interactive Virtual Environment (https://massive.ucsd.edu/ProteoSAFe/static/massive.jsp) with the dataset identifier MSV000084128. Bulk RNA-seq data presented in this study have been deposited at the Gene Expression Omnibus database (https://www.ncbi.nlm.nih.gov/geo/) with the dataset identifier GSE159926.

### Experimental Model and Subject Details

#### Hepatic fetal tissue

Human fetal liver tissues were obtained from the Human Developmental Biology Resource (HDBR) tissue bank following ethics reference 08/H0712/34+5. Sample was fixed in PFA 4% for 2 h at room temperature right after collection and embedded in OCT solution (Agar Scientific) for cryo-sectioning and staining.

#### hPSC lines

Human embryonic cell line H9 was obtained from National Stem Cell Bank, Madison, WI. BU2 hiPSC line was kindly provided from Boston University/Center for Regenerative Medicine (BU/CReM). H0-193b and H0-220c hiPSC lines were generated from human amniocytes. Briefly, human amniotic fluid (AF) was collected from patients attending the Fetal Medicine Unit or the Labour Ward of University College London Hospital (under IRAS project ID: 133888). All samples were from normal euploid pregnancies. In all cases, patients provided separate written consent. Collected samples were approved by the UK National Research Ethics Service (REC Reference number: 14/LO/0863). Each donor sample was assigned a univocal code and data were stored in a password protected NHS Database. Human amniotic fluid samples were collected, filtered using 40 μm cell strainer to remove debris/cell clumps and centrifuged at 300 g for 5 minutes. The cell pellet was re-suspended and cultured in Chang medium containing 63% α-MEM (ThermoFisher Scientific), 20% Chang Medium (Chang B plus Chang C; Irvine Scientific), 15% fetal bovine serum, FBS (ThermoFisher Scientific), 1% p/s (ThermoFisher Scientific) and 1% L-glutamine (ThermoFisher Scientific). 100mm Falcon Petri dishes (Becton Dickinson) were used for culture and incubated at 37°C in normoxic conditions. Cells were passaged at 70% confluence with TrypLE Express (ThermoFisher Scientific) and froze in freezing medium containing 90% FBS (ThermoFisher Scientific) and 10% DMSO (Sigma-Aldrich). Reprogramming to hiPSCs was performed by using a previously developed mmRNA-mediated strategy in a microfluidic platform with an integrated media distribution system (Luni et al., 2016).

#### Human primary hepatocytes

Human primary hepatocytes were purchased from BioreclamationIVT and thawed in Rat tail collagen I-coated plates with InvitroGRO CP Medium supplemented with Torpedo Antibiotic mix (all from BioreclamationIVT). The day after cells were cultured with InvitroGRO HI Medium supplemented with Torpedo Antibiotic mix for other 4 days.

### Method Details

#### Microfluidic chips fabrication

Microfluidic platforms were fabricated through standard soft-lithography techniques as reported in Giobbe et al. (2015), autoclaved and coated with 2.5% Matrigel Growth Factor Reduced, MRF (BD Biosciences) before cell seeding.

#### hPSCs-hepatic differentiation

hPSC lines were expanded in mTSR-1 (StemCell Technologies, Inc.) or StemMACS iPS-Brew XF (Miltenyi Biotech) in 0.5% MRF-coated plates, and split with 0.5 mM EDTA (ThermoFisher Scientific). Before hepatic differentiation cells were detached with TrypLE Express (ThermoFisher Scientific) and seeded as single cells in 2.5% MRF-coated 6-well plates in pluripotency medium supplemented with 10 μM ROCK inhibitor Y-27632 (Stemgent). After 48 h, pluripotency medium was removed to start differentiation.

#### Differentiation protocol #1

DE cells were derived in RPMI-1640, 1% B27 supplement minus insulin, 1% p/s, 1% Non-Essential Amino Acids (ThermoFisher Scientific), supplemented with 100 ng/mL Activin-A, 50 ng/mL Wnt3a (R&D Systems) for 1 day and with 100 ng/mL Activin-A for other 2 days. At day 3, cells were split with 1:1 surface-based ratio in either 24-well plates or microfluidic channels, previously coated with 2.5% MRF. DE cells were treated for other 7 days with knockout DMEM (ThermoFisher Scientific) supplemented with 20% knockout serum replacement, 1% p/s, 0.1 mM 2-mercaptoethanol (ThermoFisher Scientific) and 1% DMSO (Sigma-Aldrich). MH cells were obtained with L15 medium (Sigma-Aldrich) supplemented with 8.3% FBS, 8.3% tryptose phosphate broth (Thermofisher Scientific), 10 μM hydrocortisone 21-hemisuccinate, 1 μM insulin (all from Sigma-Aldrich), 2 mM L-glutamine, 1% p/s, 20 ng/ml HGF and 20 ng/ml OSM (both from R&D Systems) for 6 days.

#### Differentiation protocol #2

DE cells were derived with RPMI-1640, 1% B27 supplement minus insulin, 1% p/s, 1% Non-Essential Amino Acids, supplemented with 100 ng/mL Activin-A (R&D Systems), 20 ng/mL FGF2 (Peprotech) and 10 ng/mL BMP4 (Peprotech) for 2 days, and with only 100 ng/mL Activin-A for other 3 days. At day 5, cells were split with 1:1 surface-based ratio in either 24-well plates or microfluidic channels, previously coated with 2.5% MRF. HE cells were derived with RPMI-1640, 1% B27 supplement complete, supplemented with 10 ng/mL FGF2 (Peprotech) and 20 ng/mL BMP4 (Peprotech) for 5 days. IH cells were obtained with RPMI-1640, 1% B27 supplement complete, supplemented with 20 ng/mL HGF (Peprotech). MH cells were obtained by treating cells for 6 days with HBM basal medium supplemented with HCM single quotes (both from Lonza) and 10 ng/mL OSM.

#### Hepatic organoids formation and differentiation

IH cells for organoids formation were derived from endoderm-committed cells in μF or in CCC with the supplementation of 10ug/mL Recombinant SPARC (CSB-RP094444h, Generon), 100ug/mL bovine Fibronectin (F1141, Sigma-Aldrich), 100ug/mL Rat tail Collagen I (354236, BD) or PBS as control.

Hepatic organoids were obtained by enzymatically treating IH cells with TryplE (ThermoFisher Scientific) and re-plating 3300 cells in a 15 μL-drop of 100% MRF in hepatic organoid expansion medium (Hu et al., 2018a). Organoids were expanded as reported in Hu et al. (2018a) or differentiated to mature hepatic organoids by treating them with HBM basal medium supplemented with HCM single quotes (Lonza) supplemented with 20 ng/mL OSM (Peprotech) and 1 μM Dexamethasone (Sigma-Aldrich) for 6 days.

#### Real-time PCR analysis

Total RNA was isolated from cells with iScript RT-qPCR Sample Preparation Reagent (Biorad) solution, according to manufacturer’s instructions. Reverse transcription to cDNA was performed using the High-Capacity cDNA Reverse Transcription Kit (ThermoFisher Scientific), according to manufacturer’s instructions. Real-time PCR was performed using TaqMan Gene Expression Assay probes and Master Mix (Thermofisher Scientific) on a Step One Plus Real-Time PCR System (Applied Biosystems). *GAPDH* and *S18* were used as reference genes. All Taqman probes for quantitative RT-qPCR are listed in Table S1.

#### RNA-Sequencing and bioinformatics analysis

Total RNA was isolated with the RNeasy Micro kit (QIAGEN). Briefly, MH cells were washed once with PBS 1x and collected in 350 μL of RLT buffer at room temperature. 3 Microfluidic channels were pooled for each biological replicate. RNA was then purified according to manufacturer’s instructions. Total RNA was quantified using the Qubit 2.0 fluorimetric Assay (Thermo Fisher Scientific).

Libraries were prepared from 100 ng of total RNA using the QuantSeq 3′ mRNA-Seq Library Prep Kit FWD for Illumina (Lexogen GmbH). Quality of libraries was assessed by using screen tape High sensitivity DNA D1000 (Agilent Technologies). Libraries were sequenced on a NextSeq 500 using a high-output single-end, 75 cycles, v2 Kit (Illumina Inc.). Illumina base call (BCL) files are converted in fastq file through bcl2fastq (https://emea.support.illumina.com/content/dam/illumina-support/documents/documentation/software_documentation/bcl2fastq/bcl2fastq2-v2-20-software-guide-15051736-03.pdf) (version v2.20.0.422). Sequence reads were trimmed using bbduk software [https://jgi.doe.gov/data-and-tools/bbtools/bb-tools-user-guide/usage-guide/] (bbmap suite 37.31) to remove adaptor sequences, poly-A tails and low-quality end bases (regions with average quality below 6). Alignment was performed with STAR 2.6.0a (Dobin et al., 2013) on hg38 reference assembly obtained from cellRanger website [https://support.10xgenomics.com/single-cell-gene-expression/software/release-notes/build#mm10_3.0.0] (Ensembl 93). The expression levels of genes were determined with htseq-count 0.9.1 by using cellRanger pre-build genes annotations (https://support.10xgenomics.com/single-cell-gene-expression/software/release-notes/build#mm10_3.0.0) (Ensembl Assembly 93). We have filtered out all genes having < 1 cpm in less than n_min samples and Perc MM reads > 20% simultaneously. Differential expression analysis was performed using edgeR (Anders et al., 2015).

Data were normalized using edgeR Bioconductor package (Robinson et al., 2010) within R environment (version 3.5.1). Genes that did not have at least 0.5 count per million (CPM) in at least two samples were filtered out. Principal component analysis (PCA) was performed using median-centered log_2_(CPM+1) data with MATLAB R2017a (The Mathworks). Differentially expressed genes (DEGs) were computed with edgeR, using a mixed criterion based on false discovery rate (FDR) < 0.05 and fold change (FC) > 1.5. Hierarchical clustering of DEGs was performed on median-centered log_2_(CPM+1) data in MATLAB, using Pearson’s correlation as distance measure and complete linkage. A Volcano plot was produced, also highlighting liver genes, whose list was downloaded from Up-tissue within DAVID Bioinformatics Database (Huang et al., 2009a, 2009b). Functional enrichment analysis was performed using ReactomePA (Yu and He, 2016), with BH-corrected p value < 0.05, and ClusterProfiler (Yu et al., 2012a), with BH-corrected p value < 0.01, Bioconductor packages, and results plotted in MATLAB. Genes of secreted proteins were identified merging the results in ProteinAtlas database, based on signal peptide prediction, and Gonzalez et al. (2010) experimentally validated. Gene expression data are publicly available on Gene Expression Omnibus database GEO, https://www.ncbi.nlm.nih.gov/geo) under the GEO IDs: GSExxx.

#### SILAC experiment

H9 and H0-193 lines were adapted to TeSR-E8 (StemCell Technologies, Inc.) pluripotency medium for 3 passages before labeling for SILAC experiment. For the labeling, hPSCs were expanded for other 3 passages in SILAC pluripotency medium. This medium is the SILAC-compatible version of E8 (Chen et al., 2011), constituted by DMEM:F-12 (1:1) for SILAC (ThermoFisher Scientific) supplemented with 64 mg/L L-Ascorbic acid 2-phosphate sesquimagnesium salt hydrate (Sigma-Aldrich), 14 μg/L sodium selenite (Sigma-Aldrich), 10.7 mg/L holo-transferrin (Sigma-Aldrich), 20 mg/L insulin (Sigma-Aldrich), 100 ng/mL FGF2 (Peprotech), 2 ng/mL TGFβ (R&D Systems), 147.5 mg/L L-arginine-HCl or 151.36 mg/L ^13^C_6_^15^N_2_ L-arginine-HCl, 91.25 mg/L L-lysine-2HCl or 112.25 mg/L ^13^C_6_^15^N_2_ L-lysine-2 HCl (ThermoFisher Scientific), 800 mg/L L-proline (Sigma-Aldrich) to avoid conversion of arginine into proline (Bendall et al., 2008). Osmolarity was adjusted to 310 mOsm at pH 7.4 with HCl or NaHCO_3_.

For SILAC differentiation only protocol #2 was used, by replacing standard RPMI-1640 with RPMI-1640 for SILAC, supplemented with 147.5 mg/L L-arginine-HCl or 151.36 mg/L ^13^C_6_^15^N_2_ L-arginine-HCl, 91.25 mg/L L-lysine-2HCl or 112.25 mg/L ^13^C_6_^15^N_2_ L-lysine-2 HCl and 800 mg/L L-proline.

Conditioned media were collected every 24 h in CCC and every 12 h in μF at every medium change and stored at −80°C. HE and IH samples were generated pooling together supernatants collected from day 6 to 10, and from day 11 to 15, respectively. Cell lysates from IH cells were collected from both CCC and μF. Lysis buffer was made of RIPA Lysis and Extraction Buffer (ThermoFisher Scientific), supplemented with MS-SAFE protease and phosphatase inhibitor cocktail (Sigma-Aldrich). Total proteins concentration was quantified through Pierce BCA Protein Assay Kit (Thermofisher Scientific).

#### Sample preprocessing for LC-MS/MS and analysis

Heavy and light conditioned media were mixed in 1:1 volume-based ratio. Heavy and light lysates were mixed in 1:1 weight-based ratio. Amicon® Ultra centrifugal filters (UFC500396, Merck/Millipore) were used for protein purification and concentration. Then, proteins were reduced in 0.1M DTT at 95°C for 5 min and dissolved in 8M urea solution. Alkylation was performed for 30 min at 25°C in the dark with 55mM iodoacetamide, followed by trypsin (Promega) digestion for 16 h. Peptides were desalted by C-18 spin column (Pierce, 89870) and dried into powder. Before MS analysis, peptides were resuspended in 20 μL of 0.1% acetic acid.

Thermo Fusion Mass Spectrometer coupled with Thermo EasynLC1000 Liquid Chromatography was used to get the peptides profiles. 170 min of LC-MS gradients were generated by mixing buffer A (0.1% formic acid in water) with buffer B (0.1% formic acid in 80% ACN in water) by different proportions. Using NSI as the ion source and Orbitrap as the detector, the mass scan Range was at m/z 300-1800, and the resolution was set to 120K. The MS/MS was isolated by Quadrupole and detected by Ion trap. The activation type was HCD.

#### Proteomic bioinformatic analysis

Peak list files were searched against UniProt human reference proteome by Thermo Proteome Discoverer v. 2.2. Searches were performed using a 10 ppm precursor ion tolerance for total protein level profiling. The product ion tolerance was set to 0.8 Da in SEQUEST searches. SILAC heavy labeling on lysine (+8.014 Da, 13C(6)15N(2)) and on arginine (+10.008 Da, 13C(6)15N(4)), and the oxidation of methionine residues (+15.995 Da) were set as variable modifications. The carbamidomethyl on cysteine (+57.021 Da) was set as fixed modification. Peptide-spectrum matches (PSMs) were adjusted to a 1% and then assembled further to a final protein-level false discovery rate (FDR) of 1%. Protein quantification by iBAQ was performed using MaxQuant v.1.6.2 (Cox and Mann, 2008) and attributed to the proteins detected in Protein Discoverer according to Uniprot accession identifier. Proteins identified in only one replicate were excluded from the analysis. Common contaminants (keratins and Bos taurus proteins) were also filtered out. Correlation coefficient, histograms, Volcano and other plots were carried out using MATLAB R2017a (The Mathworks). Up- and downregulated proteins were identified by two-side one-sample t test with uncorrected p value < 0.05. Given the high similarity and sample protein overlap, to increase significance, secretome t test was performed on the merged HE and IH samples. The list of liver genes was downloaded from Up-tissue within DAVID Bioinformatics Database (Huang et al., 2009a, 2009b). ECM proteins were classified according to Naba et al. (2012). Protein interactions were downloaded from BioGrid database. Network visualization was performed using Cytoscape v.3.7 (Shannon et al., 2003). Functional enrichment analysis was performed using ReactomePA (Yu and He, 2016) and ClusterProfiler (Yu et al., 2012a) Bioconductor packages, respect to the background including all proteins identified in conditioned media and lysates of this study.

#### Immunofluorescence analysis

Cells in monolayer were fixed in 4% PFA for 10 min at room temperature and incubated in blocking solution (5% horse serum in 0.1% PBST) for 1h at room temperature. Primary antibodies were diluted in blocking solution and incubated overnight at 4°C. Secondary antibodies were diluted in blocking solution and incubated for 30 min at room temperature. Fluoroshield™ with DAPI mounting medium (Sigma-Aldrich) was used for mounting. Images were acquired on a confocal TCS SP5 microscope (Leica) at 20x and 63x magnification. For ECM proteins network analysis images were processed by ImageJ software. Original images were converted into binary and function “close” was applied. The number of junctions, branches, and meshes were analyzed through Angiogenesis Analyzer plugin. Quantification of collagen 1 deposition was performed through the “Analyze>Measure>” command in ImageJ.

Hepatic organoids were analyzed by whole-mount immunostaining. Cell Recovery solution (Corning) was used to dissolve Matrigel drops. Organoids were fixed in 4% PFA for 45 min at 4°C and incubated in blocking solution (1% BSA in 0.5% PBST) for 30 min at room temperature. Primary antibodies were diluted in blocking solution and incubated for 2 days at 4°C. Secondary antibodies were diluted in blocking solution and incubated for 1 day at 4°C. 2,2′-thiodiethanol, TDE (Sigma-Aldrich) was used for mounting.

#### Functional analysis

For ammonia detoxification assays, 2D-cultured cells or differentiated organoids were treated with 10 mM ^15^N-labeled ammonium chloride (^15^NH_4_Cl, Cambridge Isotope Laboratories) for 24h and supernatants were collected to analyze ^15^N-labeled and unlabeled urea. ^13^C,^15^N_2_-urea was added to all samples as an internal standard, and samples were derivatized in a two stage derivatization. First, urea was cyclized with 1,1,3,3-Tetramethoxypropane (Sigma-Aldrich) under acidic conditions to obtain 2-hydroxypyrimidine (2HP). 2HP was then coupled with 2,3,4,5,6-Pentafluorobenzyl bromide (Sigma-Aldrich) to yield a derivative that, upon negative ion chemical ionization gas-chromatography mass spectrometry, yields a negatively charged 2-HP fragment that includes the nitrogen and carbon atoms of the starting urea. Ions of mass/charge 95 (2HP from ^12^C,^14^N_2_-urea), 96 (^12^C,^15^N,^14^N-urea derived from ^15^N-ammonium chloride or ^13^C,^14^N_2_-urea) and 98 (^13^C,^15^N_2_-urea internal standard) were analyzed, and quantified with suitable standard curves (95/98 for unlabeled urea, 96/98 for ^15^N-urea, Figure S2E). The apparent ^15^N-urea concentration was corrected for the contribution of naturally occurring ^13^C-urea (from the ∼1% natural abundance of ^13^C) to mass/charge 96. Urea concentration is then normalized against ^15^NH_4_Cl incubation time and number of cells. For hepatic organoids, because of the difficulty in counting the number of cells in hepatic organoid culture, we used total urea to normalize the amount of labeled urea.

Human Alpha1 Antitrypsin (Abcam, ab108799) and Human Albumin (ICL Lab, E-80AL) ELISA kits were used to measure AAT and ALB secretion, respectively. The assays were performed according to manufacturer’s instruction on cell culture supernatants collected after 24h from medium change. Total urea was used to normalize the amount of secreted proteins.

Functional activities of human primary hepatocytes after 5 days in culture were assessed through Indocyanine Green (ICG) and Periodic Acid Schiff (PAS) assays as previously described (Giobbe et al., 2015). CYP3A4 activity was assessed through a P450-Glo CYP3A4 (Luciferin-PFBE) Assay (Promega) following manufacturer’s instructions. 25 μM Rifampicin (R3501, Sigma-Aldrich) was used as CYP3A4 gene inducer.

### Quantification and Statistical Analysis

Data statistical analysis was performed using Minitab v.19.2. t test was performed in Figures 1F, 1H, and 3E. One-way ANOVA was performed in Figures 4C and 4E considering ECM-treatment and μF as fixed factors, and different experiments as random factors with replicate measurements each. *n* indicates the number of replicates. Every experiment was performed at least twice with at least n = 2.

## Supplemental Information

Document S1. Figures S1–S4 and Table S1

Data S1. RNA-Sequencing Data Analysis, Related to Figure 1

Data S2. Secretome Proteomic Data Analysis, Related to Figure 2

Data S3. Lysate Proteomic Data Analysis, Related to Figure 2

Document S2. Article plus Supplemental Information
