# Supplementary material for: The Microfluidic Environment Reveals a Hidden Role of Self-Organizing Extracellular Matrix in Hepatic Commitment and Organoid Formation of hiPSCs
Source: Cell Rep. 2020 Dec 1;33(9):108453. doi: 10.1016/j.celrep.2020.108453 (PMC8237389; doi:10.1016/j.celrep.2020.108453)
Supplement: Document S1. Figures S1–S4 and Table S1 [file mmc1.pdf]

**Cell Reports, Volume *33***

## Supplemental Information

# The Microfluidic Environment Reveals a Hidden Role of Self-Organizing Extracellular Matrix in Hepatic Commitment and Organoid Formation of hiPSCs

**Federica Michielin, Giovanni G. Giobbe, Camilla Luni, Qianjiang Hu, Ida Maroni, Michael R. Orford, Anna Manfredi, Lucio Di Filippo, Anna L. David, Davide Cacchiarelli, Paolo De Coppi, Simon Eaton, and Nicola Elvassore**

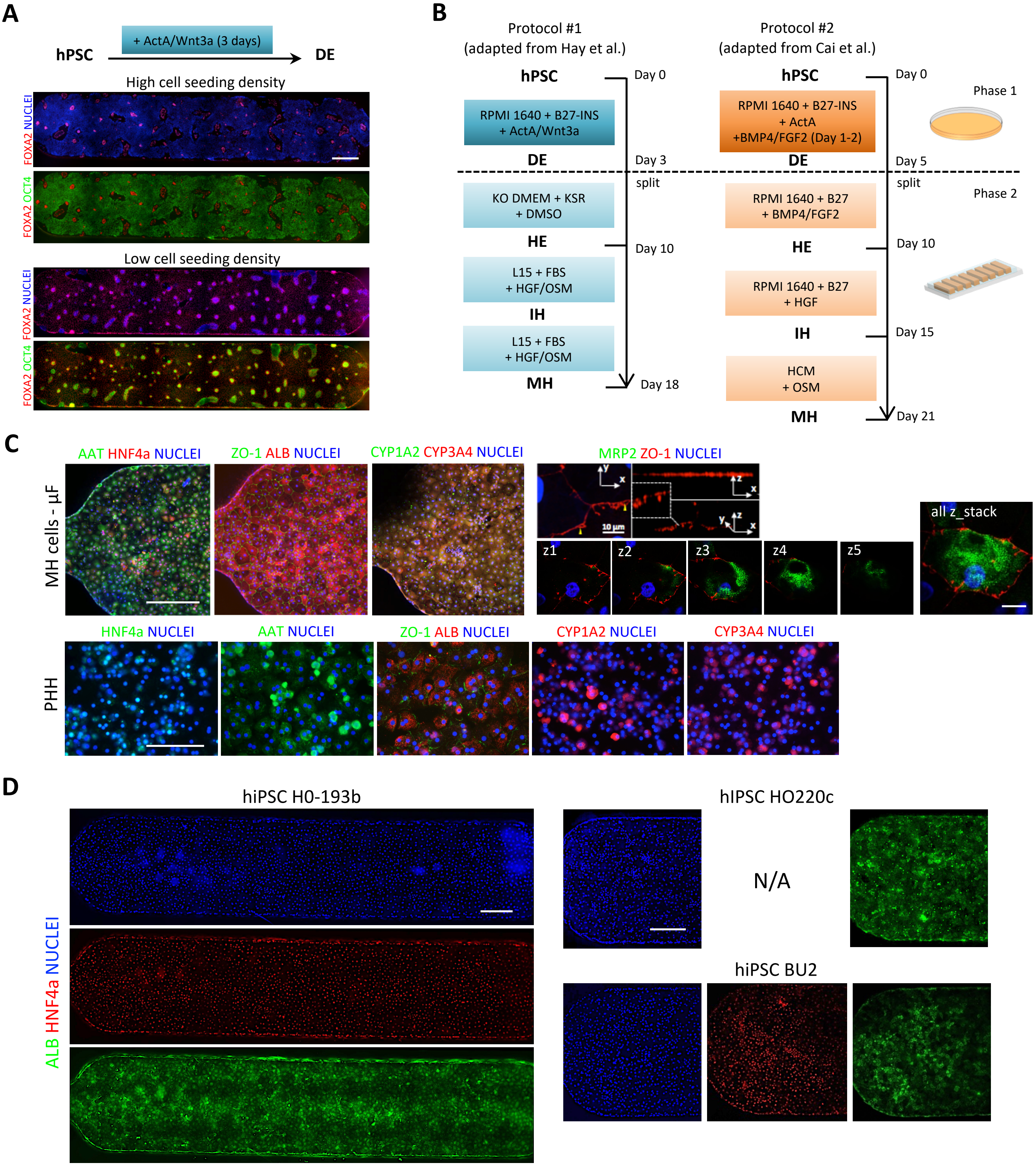


**Supplementary Figure 1: Two-phase hepatic differentiation protocol. Related to Figure 1.**
A. Endoderm commitment of hPSCs in µF with low frequency of intermittent medium change (2 times per day). Representative images of pluripotency and endoderm markers staining after 3 days of endoderm differentiation show that high seeding cell density (600 cell/mm$^2$) prevents conversion into *SOX17*$^+$/*FOXA2*$^+$ cells while retaining *OCT4* expression. Conversely, lower seeding density (300 cell/mm$^2$) allows obtaining almost homogeneous expression of endoderm markers in the microfluidic channel. Scale bar 500 µm. B. Two-phase hepatic differentiation strategy tested by adapting different published hepatic differentiation protocols. hPSCs are committed to DE cells in CCC, i.e. a standard Petri dish, during the first phase, and then injected in microfluidic channels (µF) upon cell splitting. During the second phase cells are differentiated to MH cells by maintaining a fixed low frequency of medium change (2 times per day). C. (Top-left) Representative images of hepatic markers staining of MH cells (Protocol #2) derived from H0-193 hiPSCs within the microfluidic channel. Scale bar 400 µm. (Top-right) Detail of *ZO-1* expression showing formation of bile-like canaliculi structures and expression of Multidrug Resistant protein *MRP2*. (Bottom) Expression of the same hepatic markers in Primary Human. Hepatocytes (PHH). Scale bar 50 µm. D. Representative images of *HNF4α* and *ALB* staining of MH cells (Protocol #2) along the microfluidic channels derived from H0-193, H0-220c and BU2 hiPSC lines. Scale bar 400 µm.

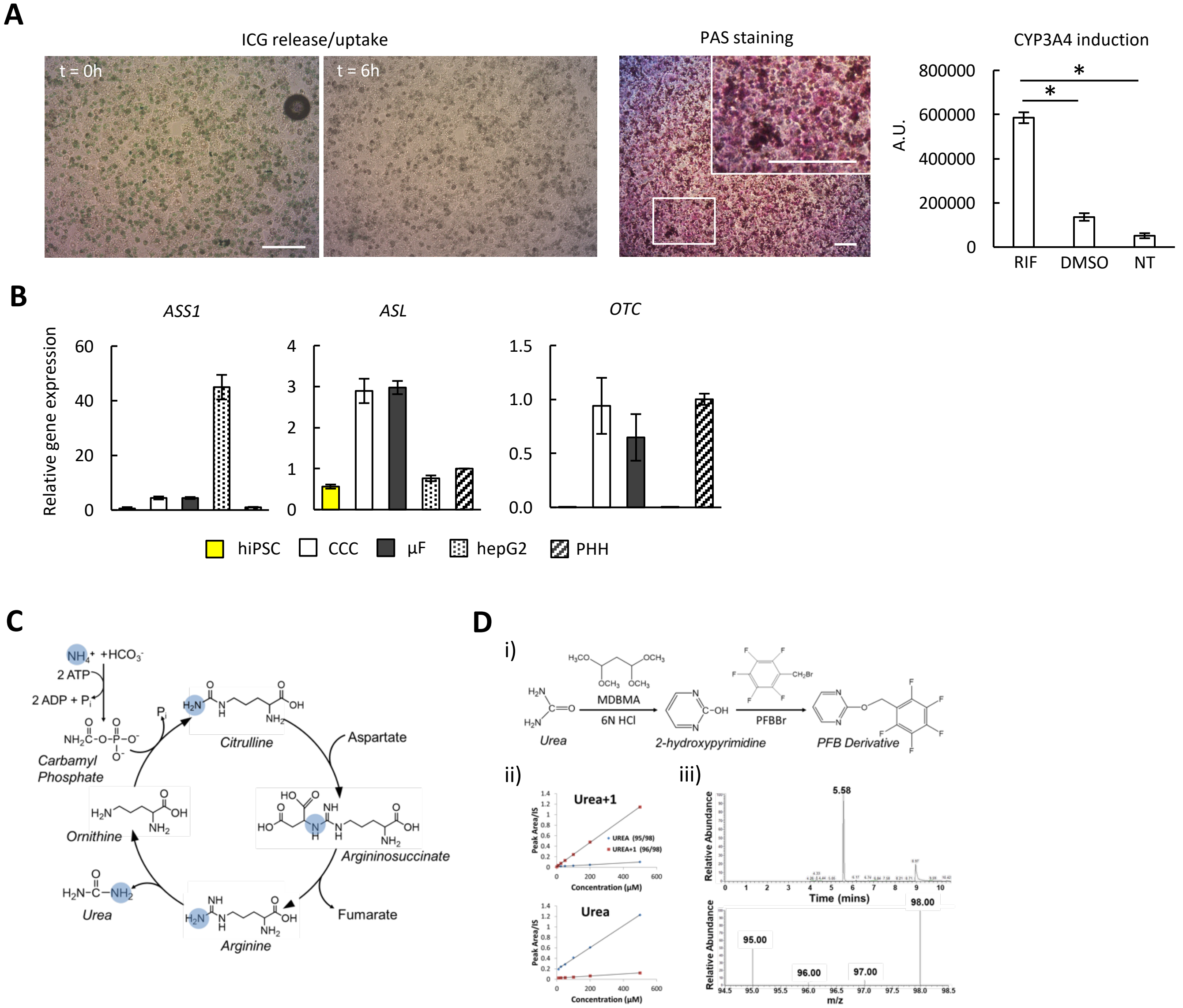


**Figure S2: Primary Human Hepatocytes (PHH) functionality and urea detoxification assay. Related to Figure 1.** A. Assessment of the functional activity of PHH through Indocyanine Green (ICG) digestion, Periodic Acid Schiff (PAS) staining and Rifampicin-mediated CYP3A4 induction. Scale bar 50 µm. Mean ± s.d., $n$=2, t-test, *$p$-value<0.05. B. Real time qPCR analysis of urea-cycle genes *ASS1*, *ASL* and *OTC* of MH cells derived in µF or in CCC from H0-193 hiPSCs. Undifferentiated hiPSCs were used as negative control. HepG2 cell line and primary human hepatocytes (PHH) were used as positive controls. Mean ± s.e., $n$=4 . C. Diagram tracing $^{15}$N-atoms from ammonium chloride (highlighted by blue circle) through the urea cycle intermediate step and ultimately into urea. This demonstrates that a single atom of $^{15}$N appears in each newly formed urea molecule, resulting in an increase in molecular weight by one amu (Urea+1). D. i) *Derivatization scheme:* Urea is first cyclized into 2-hydroxypyrimidine in a reaction with 1,1,3,3-tetramethoxypropane (MDBMA) under acid conditions. The resulting product is then reacted with Pentafluorobenzyl bromide (PFBBr) to convert it into an electron capture derivative in a phase extraction/derivatization reaction. ii) Representative standard curves generated by the analytical procedure demonstrating linearity and sensitivity of the assay. iii) Representative chromatogram and mass spectrum of a typical analytical run. The derivatized urea molecules appearing at 5.37 minutes show the following masses: Urea (m/z 95), Urea +1 (m/z 96), Urea +3 internal standard (m/z 98).

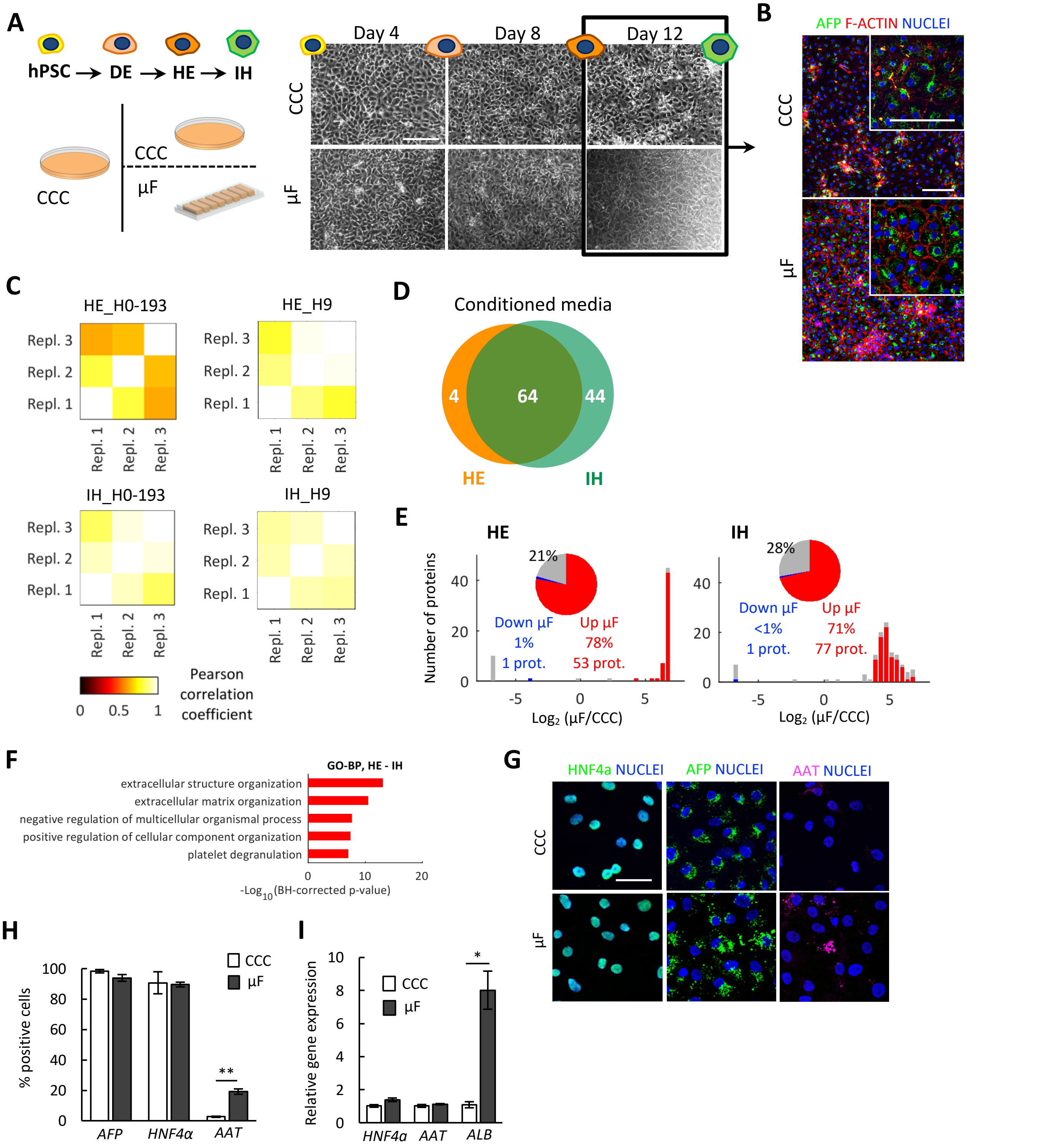


**Figure S3: hPSCs differentiation towards hepatic progenitors in μF vs. CCC. Related to Figure 2.** A. Experimental set up of hepatic differentiation towards IH cells in CCC and μF (left). Time course of hepatic differentiation of H0-193 hiPSCs through DE, HE and IH stage in CCC and μF (right). Scale bar 20 μm. B. Representative images of immunofluorescence staining at day 13 of differentiation (HE-IH stage) in CCC and in μF at different magnifications show a more defined polygonal cytoskeletal *F-ACTIN* arrangement of *AFP*$^+$ cells in μF compared to CCC. Scale bar 20 μm. C. Pearson's correlation coefficient among replicates for HE and IH samples derived from H0-193 hiPSCs and H9 hESC. D. Venn Diagram of detected proteins in conditioned media of HE and IH samples from H9 hESCs, according to the experimental set up in main Figure 3A. E. Histogram of secreted proteins detected in HE and IH samples from H9 hESCs, according to their SILAC Ratio. Proteins significantly accumulated in μF and in CCC are indicated in red and blue, respectively. The remaining detected proteins are shown in grey. Insets represent pie plots of the percentage of proteins significantly accumulated in μF in HE and IH samples. F. Functional enrichment analysis within GO-BP categories of proteins significantly accumulated in μF in HE and IH samples from H9 hESCs. G. Representative images of immunofluorescence staining of *HNF4α AFP* and *AAT* of IH cells derived in CCC and μF. Scale bar 10 μm. H. Counting of positive cells shows comparable percentage of *HNF4α*$^+$ and *AFP*$^+$ cells both in μF and CCC, and a significant higher percentage of *AAT*$^+$ cells in μF compared to CCC. Scale bar 10 μm. Mean ± s.e., *n*=4, t-test ***p*-value<0.005. I. Real time PCR analysis of hepatic-specific markers (*HNF4a*, *AAT*, *ALB*) of IH cells derived from H0-193 hiPSCs in CCC and μF. Mean ± s.e., *n*=6, t-test, **p*-value<0.01.

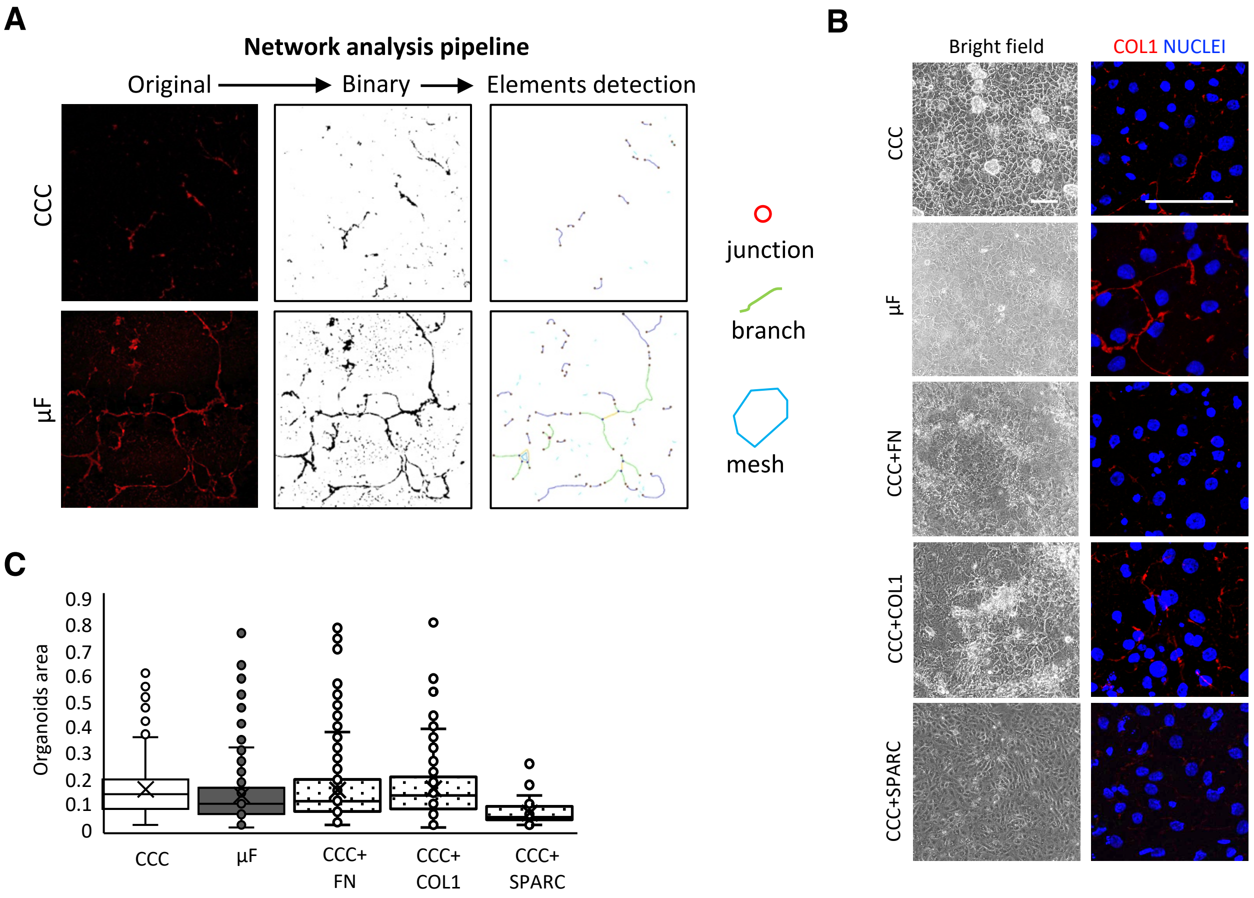


**Supplementary Figure S4: ECM remodelling quantification, COL1 deposition in ECM-treated IH cells and nascent organoids dimensions. Related to Figure 3 and 4.**

A. Analysis pipeline of ECM protein network through ImageJ software. Representative images processed for CCC and µF. B. Representative images of bright field and immunostaining analysis for COL1 of IH cells from H0-193 hiPSCs in CCC with the exogenous supplementation of 100µg/mL Rat tail COL1, 100µg/mL Bovine FN or 10µg/mL of recombinant SPARC from DE to IH stage. CCC with no treatment and µF were used as control. Scale bar 20 µm. C. Boxplot representing organoids area corresponding to organoids number in Figure 4C.

**Table S1. Related to STAR Methods. Table for Taqman probes.** List of Taqman probes used in this study.

| Probe | Source | Identifier |
|---|---|---|
| Human GAPDH (glyceraldehyde-3-phosphate dehydrogenase) | Thermofisher Scientific | Hs02786624_g1 |
| Human S18 | Thermofisher Scientific | Hs03003631_g1 |
| Human ASS1 (argininosuccinate synthase 1) | Thermofisher Scientific | Hs01597989_g1 |
| Human ASL (argininosuccinate lyase) | Thermofisher Scientific | Hs00902699_m1 |
| Human ARG1 (arginase 1) | Thermofisher Scientific | Hs00163660_m1 |
| Human CPS-1 (carbamoyl-phosphate synthetase I) | Thermofisher Scientific | Hs00157048_m1 |
| Human OTC (ornithine carbamoyltransferase) | Thermofisher Scientific | Hs00166892_m1 |
| Human ALB (albumin) | Thermofisher Scientific | Hs00609411_m1 |
| Human HNF4a (hepatocyte nuclear factor 4 alpha) | Thermofisher Scientific | Hs00230853_m1 |
| Human CYP3A4 (cytochrome P450 3A4) | Thermofisher Scientific | Hs00604506_m1 |
| Human SERPINA1 (alpha 1 antitrypsin) | Thermofisher Scientific | Hs00165475_m1 |
| Human CDH1 (E-cadherin) | Thermofisher Scientific | Hs01023895_m1 |
| Human MET (c-MET) | Thermofisher Scientific | Hs01565584_m1 |
| Human ITGA5 (integrin Alpha-5) | Thermofisher Scientific | Hs01547673_m1 |
| Human ITGA6 (integrin Alpha-6) | Thermofisher Scientific | Hs01041011_m1 |
| Human ITGB1 (integrin Beta-1) | Thermofisher Scientific | Hs01127536_m1 |
| Human ITGB4 (integrin Beta-4) | Thermofisher Scientific | Hs00236216_m1 |
| Human FN1 (fibronectin 1) | Thermofisher Scientific | Hs01549976_m1 |
